# Effectiveness of a cognitive behavioural therapy-based anxiety prevention programme for children: a preliminary quasi-experimental study in Japan

**DOI:** 10.1186/s13034-016-0091-x

**Published:** 2016-02-15

**Authors:** Yuko Urao, Naoki Yoshinaga, Kenichi Asano, Ryotaro Ishikawa, Aya Tano, Yasunori Sato, Eiji Shimizu

**Affiliations:** Research Centre for Child Mental Development, Chiba University Graduate School of Medicine, 1-8-1 Inohana, Chuo-ku, Chiba, 260-8670 Japan; Department of Nursing, Chiba Prefectural University of Health Sciences, 2-10-1 Wakaba, Mihama-Ku, Chiba, 261-0014 Japan; Department of Cognitive Behavioural Physiology, Chiba University Graduate School of Medicine, 1-8-1 Inohana, Chuo-ku, Chiba, 260-8670 Japan; Organization for Promotion of Tenure Track, General Education and Research Building (G704), University of Miyazaki, 5200 Kihara, Kiyotake, Miyazaki, 889-1692 Japan; Department of Cognitive and Behavioral Science, Graduate School of Arts and Sciences, University of Tokyo, 3-8-1 Komana Meguro-ku, Tokyo, 153-8902 Japan; Japanese Red Cross Narita Hospital, 90-1, Iidacho, Narita, 286-8523 Japan; Department of Global Clinical Research, Chiba University Graduate School of Medicine, 1-8-1 Inohana, Chuo-ku, Chiba, 260-8670 Japan

**Keywords:** Cognitive behavioural therapy, Anxiety, Prevention, Children, Adolescents, Japan

## Abstract

**Background:**

As children’s mental health problems become more complex, more effective prevention is needed. Though various anxiety and depression prevention programmes based on cognitive behavioural therapy (CBT) were developed and evaluated in Europe, North America, and Australia recently, there are no programmes in Japan. This study developed a CBT programme for Japanese children and tried to verify its effectiveness in reducing anxiety.

**Methods:**

A CBT-based anxiety prevention programme, ‘Journey of the Brave’, was developed to prevent anxiety disorders for Japanese children. Children from 4th through 6th grades (9–12 years old) in Japanese elementary schools and their parents (13 sample pairs) were the intervention group. For comparison purposes, 16 pairs were the control group. Ten weekly programme sessions and two follow-ups were conducted. Children’s anxiety levels in both groups were evaluated by child and parent self-reports using the spence children anxiety scale (SCAS) three times: pre-programme (baseline), post-programme, and 3 months following the end of the programme.

**Results:**

At 3-month follow-up, no significant difference was shown between the intervention and control groups on children’s SCAS scores in changes from baseline by using mixed-effects model for repeated measures analysis (SCAS-C: −8.92 (95 % CI = −14.12 to −3.72) and −3.17 (95 % CI = −8.02 to 1.66) respectively; the between group difference was 5.747 (95 % CI = −1.355 to −12.85, p = 0.062). On the other hand, significant reduction was shown in the intervention group on parents’ SCAS (SCAS-P) scores in change from baseline −9.554 (95 % CI = −12.91 to −6.19) and 0.154 (95 % CI = −2.88 to 3.19) respectively; the between group difference was 9.709 (95 % CI = 5.179 to 14.23, p = 0.0001).

**Conclusion:**

These preliminary results suggest this anxiety prevention programme for Japanese children was partially effective from parents’ evaluations. However, it is important to note that this study was conducted on a small sample with unbalanced groups at pre-intervention with no randomization. The positive results may require discounting due to the research limitations. A larger-scale study of the programme in elementary school classes to verify its effectiveness with a more rigorous research design is necessary.

*Trial registration*: UMIN-CTR UMIN000009021

## Background

Anxiety disorder prevalence in children and adolescents ranges between 8 and 22 % [1]. Anxiety disorders are widely recognized as the most common psychiatric disorders affecting children and adolescents [2]. The incidence rate of depression is also considerable (2.8 % in children under 13 years old and 5.6 % in adolescents between 13 and 18 years old [3]). Since mental health disorders in childhood and adolescence are believed to remit slowly and the risk of recurrence is high [4, 5], an intervention at the early symptom stages is exceedingly important to prevent problems in adulthood [6].

Preventive approaches toward children’s mental health disorder symptoms are divided into three levels [7]: (1) a universal level aiming at all children, (2) a selective level targeting an individual or a group showing some specific risk, and (3) an indicated level for those individuals or groups showing some symptoms. While each approach has its own merit, the universal approach has a number of advantages [8]. First, future symptoms in children who appeared mentally healthy at the intervention point, not just the children suffering from symptoms at that time, can potentially be prevented. This is because the universal approach tries to contribute to mental health improvement in all children. Second, this approach makes programme implementation easy, therefore allowing for ready content penetration and ease of maintaining programme effectiveness. It utilizes the school and class environments and the interaction between teachers and children. It enables repeated homework after programme completion. Third, the issue of stigma inherent in selecting only the high-risk children with symptoms can be avoided. With these merits in mind, universal level intervention is exceedingly beneficial in the execution of preventive approaches.

The development of universal-level cognitive behavioural therapy (CBT)-based preventive educational programmes and studies evaluating their effectiveness are gaining recognition in many countries. Originally, CBT was developed as a drug-free psychotherapy technique for effective treatment of mental health disorder symptoms in both children and adults. It has been positively introduced in mental health education in schools since its preventive effectiveness has been demonstrated [9]. Neil and Christensen conducted a systematic review of 27 studies in 2009 on the efficacy and effectiveness of school-based prevention and early intervention [9]. Results of the review showed that most universal, selective, indicated prevention programmes are effective in reducing symptoms of anxiety in children and adolescents with effect sizes (Cohen’s d) ranging from 0.11 to 1.37. A meta-analysis [10] reviewed the prevention of symptoms of anxiety in children and adolescents and found small but significant effects on anxiety at post-test (symptoms: g = 0.22, diagnosis: g = 0.23; SD units) and follow-up (symptoms: g = 0.19, diagnosis: g = 0.32).

The most popular version of this universal type of CBT programme for anxiety prevention in children is the FRIENDS programme developed by Barrett [11]. FRIENDS was initially developed in Australia based on the Coping Cat Programme [12] as an anxiety treatment programme [13, 14]. Subsequently, its universal level effectiveness for anxiety and depression prevention was reported in randomized controlled trials [8, 15–18].Based on this evidence, the World Health Organization started to recommend FRIENDS in 2004 as the sole children’s support programme for preventing anxiety and depression [19]. It spread globally as the content was translated into many languages. Currently, FRIENDS is implemented and studied in over 10 countries.

While the effectiveness of FRIENDS in preventing anxiety was demonstrated by the development team and in subsequent studies, several studies conducted outside Australia showed less or no positive evidence. Regarding the details of both positive and negative results of preceding studies on the FRIENDS programme, please refer to Table 1 [8, 15, 17, 18, 21–28]. The reasons for the insufficient statistical significance in subsequent studies in other countries, despite the high effectiveness initially demonstrated by the FRIENDS programme in Australia, are unclear. It is conceivable that the differences in cultural and social background between countries affected programme impact.

**Table 1:** Comparison of anxiety score reduction of various universal level FRIENDS programmes (in comparison with control groups)

Author (year)	Country	Sample (age)	N	No. of sessions time	Randomization	Instruments	Pre M (SD)	Post M (SD)	FU M (SD)	Pre-post ES (Δ)	Pre-FU ES (Δ)
Barrett and Turner (2001)	AUS	10–12 year M = 10.75	489	10 + 2 s 75 min	Randomized block design (by school)	SCAS	Teacher-I 27.00 (17.99) Psychologist-I 26.76 (15.23) C 27.44 (12.37)	Teacher-I 18.77 (14.45) Psychologist-I 19.14 (11.89) C 23.15 (13.04)		T-I 0.46 P-I 0.50 C 0.35	
Lowry–Webster et al. (2001)	AUS	10–13 year	594	10 + 2 s 60 min	Randomized block design (by school)	SCAS	I 28.09 (18.45) C 31.45 (14.76)	I 18.33 (14.07) C 28.23 (17.80)	I (12 m FU) 16.66 (13.91) C 27.54 (20.06)	I 0.53 C 0.22	I 0.62 C 0.27
Barrett, Sonderegger and Xenos (2003)	AUS	7–19 year	320	10 s 60 min	Block design (randomization not specified)	RCMAS	I-CHN 12.67 (7.63) C-CHN 9.41 (5.70) I-YUG 12.89 (7.56) C-YUG 14.75 (2.50)	I-CHN 6.50 (6.00) C-CHN 12.04 (7.28) I-YUG 6.26 (5.34) C-YUG 14.75 (2.50)		I 0.43 C 0.46 I 0.88 C 0.00	
Lock and Barrett (2003)	AUS	9–10 year, 14–16 year	733	10 + 2 s	Randomized block design (by school)	SCAS	I 22.06 (13.94) C 24.40 (12.74)	I 17.64 (12.95) C 21.26 (12.60)	I (12 m FU) 14.89 (11.64) C 17.30 (11.99)	I 0.32 C 0.25	I 0.52 C 0.56
Barrett, Lock and Farrell (2005)	AUS	9–10 year, 14–16 year	692	10 + 2 s 60 min	Randomized block design (by school)	SCAS	H I 43.41 (10.81) H C 42.32 (10.75) M I 26.18 (2.46) M C 26.91 (2.43) L I 14.85 (5.36) L C 14.34 (5.28)	H I 30.92 (10.89) H C 28.53 (12.03) M I 21.90 (10.13) M C 21.28 (9.23) L I 14.38 (8.79) L C 13.71 (8.29)	H I 21.06 (13.71) H C 26.65 (15.35) M I 17.72 (10.61) M C 18.93 (13.76) L I 11.11 (9.12) L C 12.41 (9.11)	H I 1.16 H C 0.51 M I 0.34 M C 0.39 L I 0.09 L C 0.12	H I 2.07 H C 0.58 M I 0.68 MC 0.56 L I 0.70 L C 0.37
Mostert and Loxton (2008)	ZAF	12 year	46	5 s 120 min	Quasi-experimental, nonequivalent control group	SCAS	I 42.12 (15.82) C 40.14 (12.42)	I 37.48 (16.26) C 38.05 (12.72)	I (6 m) 31.64 (16.61) C 33.71 (16.24)	I 0.29 C 0.17	I 0.66 C 0.52
Gallegos (2008)	MEX	8–13 year M = 9.9	1030	10 + 2 s 75 min	Quasi-experimental, nonequivalent control group	SCAS	I 25.82 (8.77) C 27.57 (7.95)	I 24.89 (10.18) C 26.42(10.14)	I (6 m) 22.40 (10.51) C 24.31 (10.11)	I 0.11 C 0.15	I 0.39 C 0.41
Rose, Miller and Martinez (2009)	CAN	4th 8–9 year	52	8 s 60 min	Block design (randomization not specified)	MASC	I 62.35 (17.00) C 53.65 (19.82)	I 56.88 (20.33) C 52.73 (16.50)		I 0.32 C 0.05	
Miller et al. (2011)	CAN	4th–6th M = 9.8	253	9 s 60 min	Randomized block design (By school)	MASC	I 47.10 (17.57) C 47.64 (18.51)	I 45.17 (15.25) C 42.38 (16.10)	I (17 m) 39.42 (13.40) C 36.97 (16.68)	I 0.11 C 0.29	I 0.44 C 0.58
Miller et al. (2011)	CAN	4th–6th M = 9.77	533	9 s 60 min	Randomized block design (By school)	MASC	I 45.20 (19.10) C 47.19 (17.73)	I 43.35 (20.31) C 45.61 (18.70)	I (3 m) 38.77 (17.86) C 42.10 (18.34)	I 0.10 C 0.09	I 0.34 C 0.29
Essau et al. (2012)	GER	9–12 year M = 10.91	638	10 + 2 s 60 min	Randomized block design (By school)	SCAS	I 22.53 (12.3) C 23.92 (12.2)	I 20.96 (11.7) C 23.31 (11.9)	I (6 m) 18.56 (12.2) C 24.44 (12.9)	I 0.13 C 0.05	I 0.32 C −0.04
Stallard et al. (2014)	GBR	9–10 year	1448	9 s 60 min	Cluster randomized design (By school)	RCADS30	Health-led 26.24 (15.56) School-led 24.91 (14.32) Usual-school 26.78 (16.32)		Health-led 19.49 (14.81) School-led 22.86 (15.24) Usual-school 22.48 (15.74)		I 0.43 I 0.14 C 0.26
Rodgers and Dunsmuir, (2015)	GBR	12–13 year	62	10 s 60 min	Randomized design (individual)	SCAS	I 24.68 (13.19) C 20.8 (16.5)	I 19.43 (8.97) C 19.96 (14.93)	I (4 m) 12.06 (6.91) C 16.16 (12.89)	I 0.40 C 0.05	I 0.96 C 0.28

*M* Mean; *SD* Standard deviation; *ES* Effect size; *⊿* Glass’s delta; *I* Intervention group; *C* Control group; *H* High; *M* Medium; *L* Low

Due to these considerations, an original anxiety prevention programme was developed aimed at Japanese children adapted to their individual cultural and social backgrounds.

Many previous studies indicated that anxiety occurs before depression in children [10] and the effectiveness of school-based depression prevention programmes for children is still questionable [29]. Stallard also reported a similar outcome in his 2012 study [30]. Therefore, the programme focused on anxiety rather than depression prevention. In the actual process of programme development, an existing CBT programme for anxiety disorder treatment [31–33] was used as a reference and modified for prevention purposes.

The aim of this study was to develop a CBT-based anxiety prevention programme, ‘Journey of the Brave’, for Japanese children and verify its effectiveness in a pilot study format. The hypothesis of this study is that the anxiety level of the children who participated in ‘Journey of the Brave’ will significantly reduce compared with children in the control group. If this aim was achieved, the necessary data to judge the programme efficacy should be generated and the programme feasibility would be confirmed. We would then be able to move to the next step to conduct ‘Journey of the Brave’ sessions in regular school classes as a universal approach.

## Methods

### Research design

This is a quasi-experimental study with an intervention and control group. Intervention group participants received an anxiety prevention programme and control group participants received no intervention. The main assessments were pre-programme (week 0), post-programme (week 10), and follow-up (3 months following post-programme assessment). A universal prevention study design was attempted, but higher priority was placed on programme development and execution than participant selection. As a result, some indicated level children are included in the samples giving the impression that this was an indicated prevention project.

### Programme development

Three major characteristics in ‘Journey of the Brave’ differ from FRIENDS to increase programme effectiveness in Japan.

First, the main programme content was focused on anxiety feelings and skills to deal with them. In the CBT prevention programmes developed in other countries, there are cases where depression and other psychological problems, not only anxiety, were addressed in one programme [34]. It may not be possible for children to distinguish and understand each CBT theory, resulting in failure to acquire appropriate CBT skills. In order to make CBT programmes for children more effective, it is necessary to focus on one feeling and educate them well regarding its psychological aspects, then teach them the actual CBT skill application experience. Therefore, the main objective of the programme was the understanding and acquisition of CBT skills and its theoretical basis to manage anxiety. Among CBT skills, ‘exposure’ is an especially effective CBT skill in handling anxiety problems [35]. Therefore, ‘development of anxiety hierarchy table’ and ‘exposure’ were taught carefully by developing an ‘anxiety hierarchy table’ in the first half of the sessions exposing children gradually as the programme proceeded (Table 2). A high priority was placed on children’s actual understanding through the gradual reduction of anxious feelings. In addition, two sessions were devoted to cognitive restructuring of anxiety accompanied by homework with the idea that repeated training will ensure children acquire not only behavioural but also cognitive skills. At the same time, the normalization of anxious feelings was taught carefully from the early programme stages.

**Table 2:** Outline of anxiety prevention programme

Session	Content	Study points exercise focus*
1–2	Understanding feelings of anxiety	To understand that anxiety is an important feeling in order to protect you from danger and it is not necessary to totally eliminate anxiety Clarify anxious object and set a target*
3	Body reactions and relaxation	To learn that anxiety and tension of both body and mind can be reduced by relaxation Practice and acquire techniques of breathing and muscle relaxation*
4	Anxiety level stages and stair step exposure	To learn that it is important to gradually expose self to anxiety rather than to avoid it Develop anxiety hierarchy table* Climb anxiety ladder step by step (up to Session 10)*
5	Anxiety cognition model	To learn that cognition, behaviour, and feelings are closely connected to each other and the level of anxiety changes with cognitions Develop a triangle of cognition, behaviour, and feeling*
6–7	Cognitive restructuring when anxious	To learn that anxiety can be reduced by reviewing and restructuring cognitions when anxious Restructure cognition at anxious moments*
8	Assertiveness skills to reduce social stress	To learn assertiveness skills to avoid anxiety in interpersonal relationships Study assertive ways of speaking*
9	Review	To review each session content with all participants Reviewing sessions one to eight*
10	Summary	To confirm how anxiety level and self-confidence are changed by participating in ‘Journey of the Brave’ Graduation ceremony*
11–12	Follow-up	To re-learn what was taught in each stage of the journey with all participants Reviewing sessions one to eight*

Second, in the ‘Journey of the Brave’, the main focus was interpersonal anxiety which is vital for Japanese children. It is not always effective to apply a programme used in studies in Western countries to Japanese children [36]. In order to motivate children’s interest and positive use of the programme content, it is necessary to develop and implement a programme fitting the psychological characteristics and social and cultural background of the children in the specific country. Therefore, in order to maintain children’s interest throughout the programme, an amusing story format was applied. Two likeable animal characters, one with high anxiety and the other with low anxiety, set out for a journey working on the programme together with children seeking ways to overcome anxiety. Thus, the programme was titled ‘Journey of the Brave’. Furthermore, popular animations and characters from Manga culture [37] familiar to Japanese children were utilized in the story. In order to maintain positive programme motivation, content and format must be enjoyable and fit the children’s interests and popular trends at the time and location of the presentation.

Compared with people in Western countries, Japanese are more influenced by the way they are perceived by others. Ruth Benedict, an American anthropologist, described a ‘culture of shame’ in Japan [38]. In Japan, prominent quantitative increases in anxious feelings for adolescents have been recognized in recent years. ‘The increase in severity of social phobia’ is continuing [39] and the need for programmes addressing social anxiety is high in Japanese schools. Concurrently, school is the main forum for learning social skills. Therefore, consideration of children showing high social anxiety is required in implementing an anxiety prevention programme at Japanese schools. In order for children with high social anxiety to work on the programme comfortably, group work format between children was completely avoided. In the individual work format, each child dealt with his or her own problem and took notes in individual workbooks. With these considerations, it was easier for each child to face his or her own problem even in the session room. In addition, assertive communication is taught in session 9, handling the interpersonal anxiety issue directly.

Third, the programme is custom tailored to fit the Japanese school scene for both teachers and children. There are severe time constraints in Japanese elementary schools; one class session cannot exceed 45 min since each class is supposed to finish within 1 h including 15-minute breaks between classes. In addition, one teacher teaches all curriculum subjects in his or her class and teaching assistants are simply not available. Therefore, in the ‘Journey of the Brave’, the programme was modified to fit Japanese schools. For example, each programme session content was reduced to fit a 45-minute class and a manual was prepared for teachers to be able to conduct sessions following the manual content without an assistant.

### Participants

Because this was a pilot study, participants were recruited through poster advertisements at various public facilities in City A, targeting 9–12-year-old elementary schoolchildren and their parents. Out of 24,000 same age children in the city, thirteen participants going to public elementary schools answered the advertisements and were identified as the intervention group. Sixteen children of the same age were selected as the control group. Parents of both groups agreed to sign the consent forms.

Ideally, the same method of recruitment should have been used in both groups. However, since there were insufficient responses to the control group recruitment advertisement, a snowball sampling method was used for this group. The snowball samples were recruited through the researcher’s network. Three parents were asked to find parents of children in the same age category.

Although specific exclusion criteria were not applied, physically or developmentally disabled children were automatically excluded by limiting the sample to children grades 4–6 (ages 9–12) going to public school in Japan. Children with disabilities typically enter elementary schools specially designed for them in Japan.

### Procedure

Ten weekly 60-minute sessions including 15-minute breaks were conducted with the intervention group children at a community centre meeting room after school between April and June 2013. For each session, PowerPoint slides, a workbook, and a homework sheet were prepared. The programme contents were supervised by a MD/PhD university professor who is a CBT expert. Each session consisted of a 45-minute presentation conducted by the first author (YU) who is a psychiatric nurse and developed the programme. At least one clinical psychologist (RI or AT) attended each session as an observer/assistant. Each session proceeded with one project workbook page on the screen and a workbook on each participant’s desk. A session summary for parents was distributed each time. At the end of each programme session, a homework assignment was given in order to comprehend and consolidate the programme content; the finished homework was returned at the next session. Additionally, two 60-minute parents’ meetings to explain the procedure and programme content were held the mornings of the programme period weekends after sessions 5 and 9, respectively. Anxiety levels of intervention group children were measured at the session location and other scores were taken at their homes.

### Measurement

The outcome measure was children-and parent-reported child anxiety symptoms, as measured on the Spence children’s anxiety scale (SCAS) [40], because it was one of the most valid measurements for assessing child anxiety matching the diagnostic standard. SCAS scores range between 0 (never) and 3 (always) and the maximum possible score of the 38 anxiety items is 114. According to a previous study, average SCAS score of 7- to 12-year-old children was 20.51 (SD = 14.20) and the cut-off point was 42 [41].

SCAS-Child version (SCAS-C) was used to assess child-reported anxiety symptoms and the corresponding SCAS-Parent version (SCAS-P) was administered to parents. Each measure contains 38 items regarding children’s anxiety symptoms with six subcategories: separation anxiety, social phobia, panic disorder/agoraphobia, generalized anxiety disorder, physical injury fears, and obsessive–compulsive disorder. The questions are applicable to 8- to 15-year-old children. Both measures have good psychometric properties [42] and the internal consistency for the current sample was acceptable (child version, α = 0.92 [40]; parent version, α = 0.89 [43]). Good reliability and validity of the Japanese versions of the SCAS have been reported [44].

### Statistical analysis

For the baseline variables, summary statistics were constructed using frequencies and proportions for categorical data and means and SDs for continuous variables. The patient characteristics were compared using Fisher’s exact test for categorical outcomes and *t* tests or the Wilcoxon rank sum test for continuous variables, as appropriate.

Primary analysis was performed using the mixed-effects model for repeated measures (MMRM) with treatment group, time (week), and interactions between treatment group and time (week) as fixed effects; an unstructured covariate was used to model the covariance of within-subject variability. MMRM analysis used all available data and assumed that any missing observations were missing at random. Under the ignorable missing data framework, MMRM analysis appears to be a robust approach in estimating the true treatment difference and in controlling Type I error rates [45, 46]. However, in the case of data that are not missing at random, these inferential techniques valid for missing-at-random data are typically no longer valid [47, 48].

All statistical tests were two-tailed and a p of .05 was employed. Effect sizes and 95 % confidence intervals (CI) were calculated using R 3.1.1. [49] and other statistical analyses were performed with IBM SPSS Statistics for Windows, Version 19.0 (IBM, Armonk, New York, USA) and SAS software version 9.4 (SAS Institute, Cary, NC, USA).

## Results

The differences in participant characteristics, gender, and age were analysed between the 13 intervention group and 16 control group children at pre-test. There were no significant differences (Table 3). Next, in order to compare the *group* differences in baseline SCAS scores at pre-test, t-tests were conducted. There were no significant differences on SCAS-C but there were significant differences in SCAS-P scores (P = 0.002; Table 3).

**Table 3:** Participants’ demographic data and baseline SCAS score

	Intervention (n = 13)	Control (n = 16)	p value
Gender female	6 (46 %)	3 (19 %)	0.58
Male	7 (54 %)	13 (81 %)	
Grade 4th	5 (38 %)	6 (38 %)	0.87
5th	3 (24 %)	5 (31 %)	
6th	5 (38 %)	5 (31 %)	
SCAS-C	20.62 (14.45)	18.56 (9.94)	0.66
SCAS-P	21.08 (11.15)	9.38 (7.42)	0.002

*SCAS-C/P* Spence children’s anxiety scale-child/parent versions

**Fig. 1:**
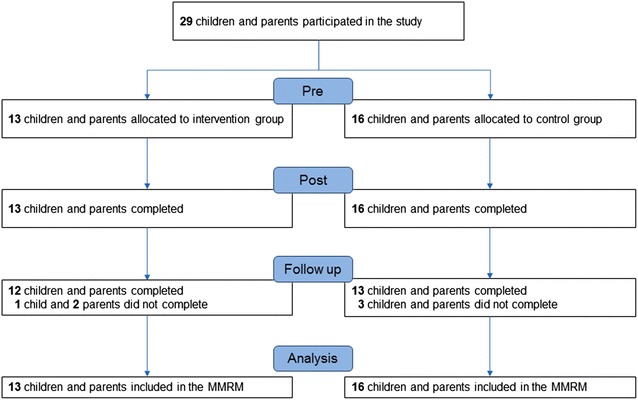
Flow-chart shows the number of children and parents at each time and a sample count of MMRM. MMRM, mixed-effect model for repeated measures

Out of 156 session opportunities (13 participants times 12 sessions), there were only eight absences (95 % attendance). Although the number of respondents at post-test (13 intervention group and 16 control group) remained the same, one intervention group family and three control group families did not return the questionnaire (Fig. 1).

**Fig. 2:**
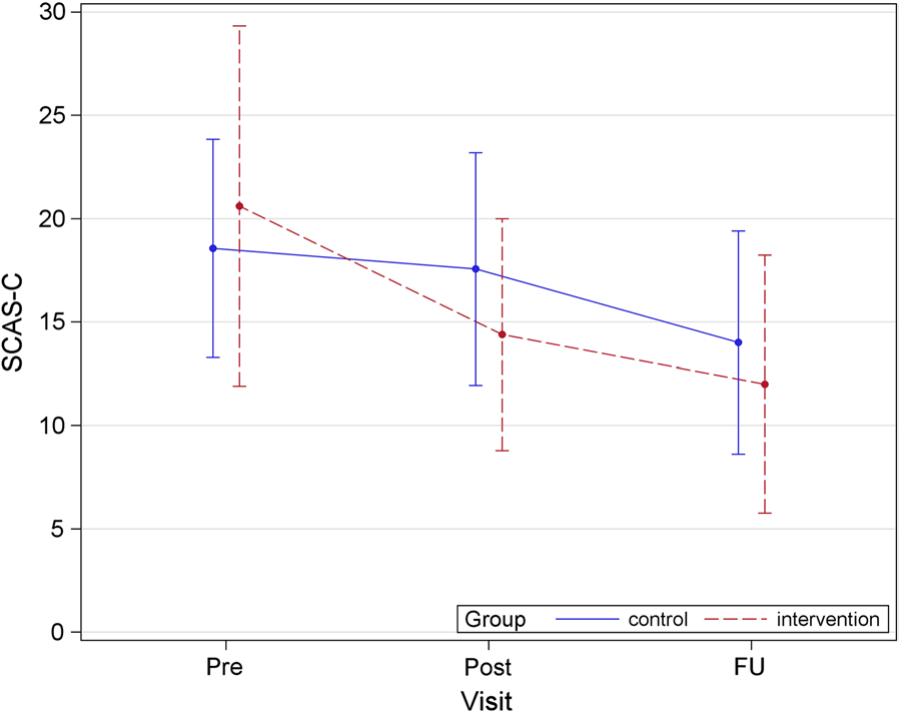
Mean total SCAS-C scores in each group during study shows average SCAS-C scores of the intervention group and the control group for each time period. SCAS-C, Spence children’s anxiety scale-child version

**Fig. 3:**
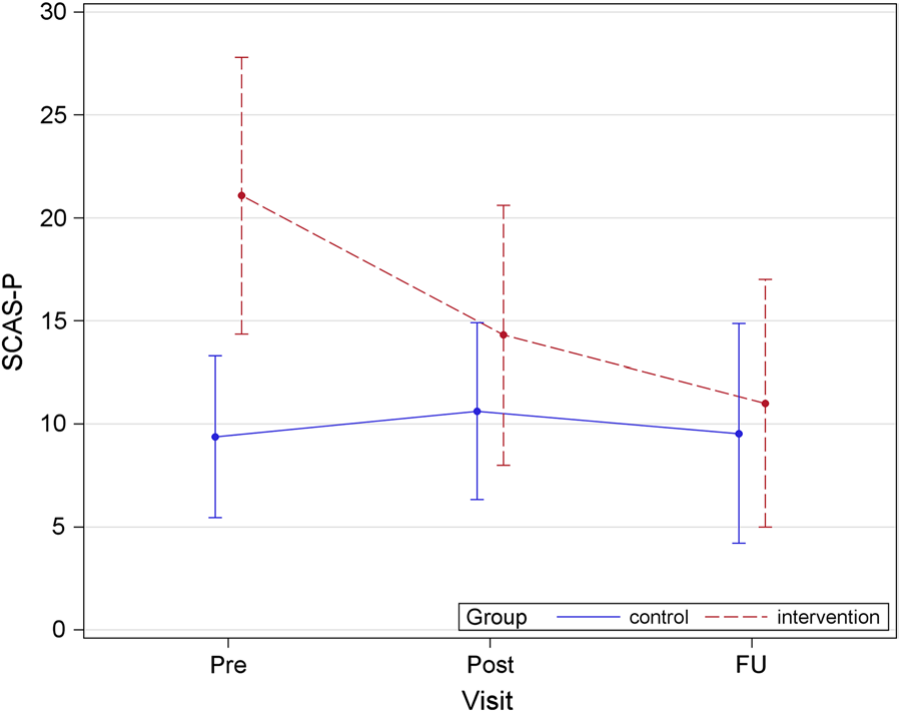
Mean total SCAS-P scores in each group during study shows the SCAS-P scores of the intervention group parents and the control group parents for each time period. SCAS-P, Spence children’s anxiety scale-parent version

After 10 weeks, the adjusted means of SCAS-C were 14.38 (95 % CI 8.87–19.89) in the intervention group and 17.56 (95 % CI 12.59–22.53) in the control group. At week 23, the adjusted means were 11.77 (95 % CI 6.69–16.84) and 14.97 (95 % CI 10.27–19.67), respectively (Fig. 2 and Table 4). In primary analysis, at the 3-month follow-up time point, estimated mean changes in SCAS-C from baseline by MMRM analysis were −8.92 (95 % CI −14.12 to −3.72) and −3.17 (95 % CI −8.02 to 1.66) for the intervention and control groups, respectively; the group difference was 5.747 (95 % CI −1.355 to −12.85, p = 0.062). On the other hand, after 10 weeks, the adjusted means of SCAS-P were 14.31 (95 % CI 9.24–19.37) in the intervention group and 10.62 (95 % CI 6.06–15.18) in the control group. At week 23, the adjusted means were 11.50 (95 % CI 6.53–16.47) and 9.51 (95 % CI 5.02–14.00), respectively (Fig. 3 and Table 4).

**Table 4:** Estimated values and changes from baseline at each visit in SCAS-C and SCAS-P by MMRM

Score	Visit	Intervention (n = 13)	Control (n = 16)	Between group difference for baseline change	p value
		Estimated mean (95 % CI)	Estimated mean (95 % CI)		
SCAS-C	Pre	20.61 (13.94–27.28)	18.56 (12.54–24.57)	NA	NA
	Post	14.38 (8.87–19.89)	17.56 (12.59–22.53)	5.231 (−0.176–10.64)	0.057
	FU	11.77 (6.69–16.84)	14.97 (10.27–19.67)	5.747 (−1.355–12.85)	0.108
SCAS-P	Pre	21.07 (15.99–26.16)	9.37 (4.79–13.95)	NA	NA
	Post	14.31 (9.24–19.37)	10.62 (6.06–15.18)	8.019 (4.284–11.75)	0.0002
	FU	11.50 (6.53–16.47)	9.51 (5.02–14.00)	9.709 (5.179–14.23)	0.0002

*MMRM* mixed-effect model for repeated measures; *SCAS-C/P*, Spence children’s anxiety scale-child/parent versions; *FU* follow-up; *NA* not available

In primary analysis, mean reductions in SCAS-P from baseline were −9.554 (95 % CI −12.91 to −6.19) and 0.154 (95 % CI −2.88 to 3.19) for the intervention and control groups, respectively; group difference was 9.709 (95 % CI 5.179 to −14.23, p = 0.0001).

In addition, participants’ evaluation forms were filled at the end of the 10th session by every participant as well as parents. The evaluations of both children and parents overall were quite positive and there were no negative evaluations.

## Discussion

This study developed a CBT-based anxiety prevention programme that would be effective for Japanese children and studied its feasibility as well as possible execution difficulties with a small sample trial to verify its effectiveness. Initially, there was a concern whether all of the intervention group children would be able to complete the programme because it was necessary for them to commute to the city facility once every week after school for a period of 2.5 months. However, there was absolutely no halfway dropout. Thus, we believe the feasibility of our programme was partially confirmed by this fact.

Significant anxiety reduction was demonstrated only by the parents’ evaluations. No statistically significant interaction was demonstrated between groups in children’s evaluations. It is regrettable that SCAS-C scores (the primary outcome measure of this study) did not significantly reduce and our original hypothesis was not proven. Considering that most of the preceding studies’ evaluations (Table 1) were completed by the children and many showed evidence of intervention effectiveness, it is regrettable that our study did not show positive results in between group comparisons of children’s evaluations even though positive reduction was shown in the intervention group. Concurrently, however, anxiety reduction was shown by parents’ evaluation. Therefore, the programme was proven neither effective nor ineffective at this stage. The following is our thoughts on the results of SCAS-C and SCAS-P and study limitations.

### SCAS-C

As mentioned previously, positive anxiety score reduction was regretfully not shown by the children’s self-evaluations between group comparisons. The reason is not clear yet, but it is necessary to continue to improve the research method as well as the programme content. One possible reason for this result is that children’s own anxiety standards may have changed between the pre-programme and post-time periods. For example, in answering the statement ‘I feel scared if I have to sleep on my own’, if children answered ‘often’ before the programme, there is the possibility that they gave the same answer ‘often’ even if they started to sleep alone after the programme due to the learned exposure. This is one limitation of questionnaire-based studies; therefore, it may be necessary to conduct interview-based evaluations concurrently in the future.

This programme was based on CBT content used to treat anxiety disorder and converted to prevention purposes. There is a possibility that some children did not fully understand the session content and were unable to use the acquired skills since each class was conducted in a group format without detailed attention given to each child’s own level of understanding. It may be necessary to evaluate the level of CBT understanding and achievement of each participant more carefully in the future. We wish to confirm this point through a universal approach trial in the future.

### SCAS-P

In this study, parents were asked to evaluate their children’s anxiety reduction. Parents observe children daily and are in a position to evaluate the children’s behaviour objectively. Therefore, if parents reported that anxiety was reduced in their children, it may partially be the result of this programme.

Concurrently, however, the programme participants were recruited by advertising. There is a strong possibility that the parent saw the advertisement and decided to have their children participate. If this is the case in the sampling, there is a possibility that the expectation levels of the parents making the decision were high initially and tended to overestimate programme effectiveness. In the future, it will be necessary to conduct interview-type research surveying specific changes in children leading to concrete anxiety reduction in addition to SCAS-P evaluation.

Moreover, it should be noted that there was a significant difference in SCAS-P at pre-programme baseline in this study and this fact may have contributed to the result. It would be better to minimize this type of bias in conducting subsequent studies and improve parents’ evaluation methods in the next stage.

## Limitations

There are several serious shortcomings in the research design [50] of this study. First, there is an issue of sampling. Theoretically, in designing a programme effectiveness study aimed at universal level usage in schools, participants should be recruited from school classes. However, in conducting this study, advertising was used initially since it was a more practical and realistic approach for a pilot study. Japanese teachers and schoolmasters are very conservative and it was not likely that they would accept a universal level pilot study trial with no success history in their school classes. It was more persuasive to demonstrate some effectiveness first before making official presentations to various schools for full-scale participation.

It is conceivable that this may have attracted children with higher anxiety levels; although the original intention was to conduct a universal trial, it may appear that this was an indicated prevention level project. Two groups were recruited by different methods and there was no randomization in the groups. It is necessary for programme effectiveness verification to conduct the programme sessions in regular school classes. This was impossible because of various constraints in this study and universal level execution was abandoned. In addition, although the advertising method was originally applied for both groups’ recruiting, there were minimal responses for control group candidates and the snowball method was used for this group. It is natural that the parents of the intervention group who were recruited by advertising showed higher pre SCAS-P scores than the control group’s parents who were recruited by the snowball method.

This study is positioned as a preliminary study before full implementation in school classes. Although statistical significance was demonstrated in the intervention group, the small sample size made it difficult to generalize the results. With these sampling limitations, a major imbalance of SCAS**-**P scores emerged. The biased influence of a statistically significant high pre SCAS-P score in the intervention group parents compared with the control group that may have contributed to the result should be seriously considered. In other words, the positive interaction result of SCAS-P shown from parents’ evaluations of their children’s anxiety score reductions at post- and FU compared with the control group parents’ evaluations may have been due to the pre SCAS-P being significantly higher; the positive result should accordingly be viewed cautiously.

In verifying the effectiveness of this programme with stronger evidence in the future, it is necessary to conduct the process under much more rigorous research design recruiting a universal level of participants from the regular school system. Both the intervention and the control groups would be randomized for even sample distribution.

In addition, there are other possible limitations such as the single usage of the SCAS to estimate symptoms as the evaluation tool for anxiety reduction. Moreover, the follow up data is only three months post-programme. It cannot be said definitely that children’s anxiety was prevented because children’s anxiety levels were lower immediately following the program. Therefore, in order to firmly secure evidence of long-term anxiety prevention, it is necessary to demonstrate the long-term effectiveness of this programme clearly by using longer follow-up periods [10, 51] and to conduct cohort research analysing the prevalence rate of mental health disorders such as anxiety disorders or depression.

Finally, although one author conducted the program sessions in this study, there is a possibility that effectiveness differs depending on who executes the program [51, 52]. In order to integrate the universal approach into the regular school system in Japan in the future, it would be necessary to estimate the effectiveness of the schoolteachers conducting the sessions. Unless proven evidence of meaningful effectiveness can be expected by whoever conducts the session, it would be difficult to disseminate the program widely throughout Japanese schools.

Therefore, a programme that is easy for teachers to manage at school is being planned and a training manual is being prepared so that any teacher can execute the program. Finally, programme effectiveness based on the school trial sessions will be evaluated.

## Conclusions

The preliminary results suggest this anxiety prevention programme for Japanese children was partially effective from parents’ evaluation. However, it is important to note that this study was conducted on a small sample with unbalanced groups at pre-intervention with no randomization. The positive result may need to be discounted by the research limitations. A future larger-scale study is necessary to execute the programme in elementary school classes and verify its effectiveness with more rigorous research design.

## Abbreviations

CBT: cognitive behaviour therapy
CI: confidence interval
FU: follow-up
SCAS-C/P: spence children’s anxiety scale-child/parent versions
MMRM: mixed-effect model for repeated measures
